# Exploring the Low Force-High Velocity Domain of the Force–Velocity Relationship in Acyclic Lower-Limb Extensions

**DOI:** 10.1186/s40798-023-00598-0

**Published:** 2023-07-13

**Authors:** Jean Romain Rivière, Jean-Benoît Morin, Maximilien Bowen, Matt R. Cross, Laurent A. Messonnier, Pierre Samozino

**Affiliations:** 1 Laboratoire Interuniversitaire de Biologie de La Motricité, Univ Savoie Mont Blanc, EA 7424, 73000 Chambéry, France; 2 Laboratoire Interuniversitaire de Biologie de La Motricité, Université Jean Monnet Saint-Etienne, Lyon 1, Université Savoie Mont Blanc, 42023 Saint-Etienne, France; 3 Sports Performance Research Institute New Zealand (SPRINZ), Auckland University of Technology, Auckland, New Zealand

**Keywords:** Horizontal leg-press, Ballistic movements, Hill’s equation, Fenn and Marsh’s equation, Force production capabilities, Strength

## Abstract

**Purpose:**

To compare linear and curvilinear models describing the force–velocity relationship obtained in lower-limb acyclic extensions, considering experimental data on an unprecedented range of velocity conditions.

**Methods:**

Nine athletes performed lower-limb extensions on a leg-press ergometer, designed to provide a very broad range of force and velocity conditions. Previously inaccessible low inertial and resistive conditions were achieved by performing extensions horizontally and with assistance. Force and velocity were continuously measured over the push-off in six resistive conditions to assess individual force–velocity relationships. Goodness of fit of linear and curvilinear models (second-order polynomial function, Fenn and Marsh’s, and Hill’s equations) on force and velocity data were compared via the Akaike Information Criterion.

**Results:**

Expressed relative to the theoretical maximal force and velocity obtained from the linear model, force and velocity data ranged from 26.6 ± 6.6 to 96.0 ± 3.6% (16–99%) and from 8.3 ± 1.9 to 76.6 ± 7.0% (5–86%), respectively. Curvilinear and linear models showed very high fit (adjusted *r*^2^ = 0.951–0.999; SEE = 17-159N). Despite curvilinear models better fitting the data, there was a ~ 99–100% chance the linear model best described the data.

**Conclusion:**

A combination between goodness of fit, degrees of freedom and common sense (e.g., rational physiologically values) indicated linear modelling is preferable for describing the force–velocity relationship during acyclic lower-limb extensions, compared to curvilinear models. Notably, linearity appears maintained in conditions approaching theoretical maximal velocity. Using horizontal and assisted lower-limb extension to more broadly explore resistive/assistive conditions could improve reliability and accuracy of the force–velocity relationship and associated parameters.

## Key Points

- Lower-limb extensions performed horizontally with assistance resulted in very low inertial and resistive conditions which provides access to assessment conditions approaching neuromuscular limits (i.e., near theoretical maximal velocity) in acyclic lower-limb extensions.
- Compared to curvilinear models, linear modelling of the force–velocity relationship in acyclic lower-limb extensions displayed the best combination of fitting the underlying data, complexity of the modelling approach, and physiologically rational output parameters.
- Researchers and practitioners can confidently use linear modelling to describe the force–velocity relationship in acyclic lower limb extensions up to 75% of maximum lower-limb extension velocity in average, and up to 85% for some individuals.

## Background

Ballistic movements are common in daily life and crucial in many sports. Success during such maximal efforts relies on high force and power production over the entire movement. Human dynamic maximal force and power generation capabilities depend on movement velocity and are well described by the force–velocity (*F–v*) and power-velocity (*P*–*v*) relationships [e.g., 1]. These two relationships have four main output variables of interest: (i) *P*_max_, the apex of the *P–v* relationship representing the maximal power that can be reached at a specific velocity, called optimal velocity (*v*_opt_); (ii) *F*_0_, the force-intercept of the *F–v* relationship, corresponding to the theoretical maximal force produced at zero velocity; (iii) *v*_0_, the velocity-intercept of the *F–v* relationship, corresponding to the theoretical maximal velocity until which force can be produced, and (iv) the slope (or curvature) of the *F–v* relationship representing the rate at which force production capabilities decrease when velocity increases. *F*_0_ and *v*_0_ represent strength indexes of force production capabilities at low and high velocities, respectively, i.e., in the high force-low velocity and low force-high velocity domains of the *F–v* relationship. *F–v* and *P–v* relationships have seen wide adoption in testing and training of ballistic performance. For instance, biomechanical modelling [2–4] and experimental results [5] indicate that ballistic performance depends on both *P*_max_ and the slope of the *F–v* relationship. Several studies have provided a basis for training guidelines [6], which revolve around individualization and subsequently improved training efficiency [e.g., 7–9]. Consequently, the *F–v* relationship interests both practitioners and coaches.

In lower-limb ballistic extensions, *F–v* and *P–v* relationships can be evaluated via i) cyclic extensions, such as during running [e.g., 10] or cycling [e.g., 11], and with ii) acyclic extensions, like during vertical [12] and horizontal jumping [e.g., 13] or on inclined/horizontal leg-press devices [e.g., 3, 14]. While cyclic extensions involve force orientation technique, and thus their transferability is limited, acyclic extensions rather consider the quasi-total external force developed by lower limbs, assessing less exercise-specific strength indexes. Once collected, mathematical modelling is used to determine the *F–v* relationship, from which the variables of interest are extracted (i.e., *F*_0_, *v*_0_, *P*_max_ and the slope). In the case of lower-limb acyclic extensions, the *F–v* relationship has been mostly described using linear modelling [e.g., 13, 15, 16]. The linear model is based on the basic first-order polynomial function and typically exhibits very high goodness of fit (GoF; i.e., high coefficient of determination [*r*^2^] and low standard error of estimate [SEE]) on force and velocity data. Nevertheless, the use of linear models has been questioned [e.g., 17–19] since the *F–v* relationship’s evaluation typically includes force measurements across a restricted range of velocity conditions (~ 20 to ~ 50–60%*v*_0_) [e.g., 13, 16, 17]; accordingly, because more than half of the *F–v* relationship is typically undescribed by experimental data, any linearity observed might instead represent a partial range of an overall curvilinear shaped relationship. Two empirical arguments have been proposed to support the use of curvilinear models in acyclic lower-limb extensions. Firstly, in mono-articular human movements or single-muscle in vitro conditions [20, 21], curvilinear models fit a wide range of velocity conditions (from ~ 0 to ~ 75–99%*v*_0_). Under these conditions, curvilinear models were based on i) an exponential function (*F&M's*_Eq_; [20]); ii) a reciprocal function (rectangular hyperbola, *Hill’s*_Eq_; [21–23]); or iii) a combination of the two [24], which showed very high GoF and SEE on force and velocity data. The second argument was that the basic second-order polynomial (*Poly*_2_) or exponential functions typically exhibit higher GoF compared to the one of the linear model, when fitted to data situated within the typical restricted range of velocity [e.g., 17, 18, 25]. Of note, before studying *F–v* relationship on isolated muscles, Hill and colleagues studied it on single (elbow flexion; [26]) and multi-joint (pedalling; [27]) movements, using linear model. Several experimental studies have explored the *F–v* relationship beyond the typical 20–60%*v*_0_ range and assessed the GoF of functions of the linear and curvilinear models (n.b., from this point on, fitting quality [i.e., *r*^2^ and SEE] of the function of a model will be discussed directly as fitting quality of a model).

In the high force-low velocity domain (i.e., from 0 to ~ 20%*v*_0_), researchers typically agree that linear models simply and accurately fit force and velocity experimental data and estimate *F*_0_ [e.g., 25–28]. In the low force-high velocity domain (i.e., from ~ 60 to 100%*v*_0_), only three studies have explored lower-limb force production in conditions nearing maximal velocity [14, 29, 30]. Yamauchi et al. [14] reported higher GoF of the linear model than curvilinear models (using the basic exponential function) up to ~ 97%*v*_0_. However, force and velocity were collected at specific joint angles as peak values, which limits the transferability of the results to other experimental conditions [14, 31]. Lindberg et al. [30] reported very high GoF of the linear model until ~ 85%*v*_0_, without considering curvilinear models in their analyses. Alcazar et al. [29] observed the *F–v* relationships of some participants were better described by the linear model and others by a curvilinear model (using *Hill’s*_Eq_), although the underlying force and velocity data in the low force-high velocity and the high force-low velocity domains were obtained via different exercise conditions and analyses. In addition to these three studies, two empirical arguments support adopting a ‘simpler’ linear model: firstly, Bobbert [32] showed that the *F–v* relationship displayed a “quasi-linear” shape from ~ 5 to 90%*v*_0_ in a simulation of lower-limb extensions performing a leg-press task (each muscle’s *F–v* relationship was described by *Hill’s*_Eq_), and; secondly, the *F–v* relationship has been described by the linear model in other multi-joint movements, such as during cycling and running, with high GoF on experimental data covering the wide range of ~ 20–90%*v*_0_ [11, 33]. In any case, while ultimately there is no consensus (likely owing to a dearth of research exploring low force-high velocity domains), the current evidence indicates linear modelling likely best describes the *F–v* relationship.

Previous studies have typically evaluated models by detecting significant differences between GoF, only comparing non-adjusted *r*^2^ and SEE. This is problematic as these two indexes naturally inflate with models’ complexity, but without penalizing the use of higher degrees of freedom, favoring thus more complex models. Moreover, should a better fit be detected, it is impossible to clarify whether adding degrees of freedom describes the experimental data well enough to justify their higher complexity over simpler models. Indeed, more degrees of freedom increases variance, which can lead to noise in the model fit and biased or physiologically illogical estimations of outputs (e.g., *F*_0_ or *v*_0_; [25]). Hence, previous works did not consider the principle of parsimony, which dictates that *“Numquam* *ponenda* *est* *pluralitas* *sine* *necessitate”*, as stated by William of Ockham (transl. plurality must never be posited without necessity; [34]). Applied here, models with higher degrees of freedom should not be preferred when simpler models are equally experimentally and statistically evidenced [35], as recommended in sport and exercise science [36].

In this study we aimed to compare the accuracy and relevance of linear and curvilinear models to describe the force–velocity relationship in acyclic lower-limb extensions across a broad range of velocity conditions. We hypothesized that, despite higher GoF of curvilinear models, their greater complexity would not improve the description of force and velocity data to an extent that would warrant their use instead of the simpler linear model.

## Methods

### Participants

Nine healthy participants (8 males and 1 female, age = 21.3 ± 0.5 years, mass = 70.6 ± 9.1 kg and stature = 1.78 ± 0.07 m) gave their written informed consent to take part in this study, which was approved by the local ethics committee and complied with the standards of the declaration of Helsinki. All participants practiced regular physical activities (strength and endurance training) with no common training program between them (in terms of volume and intensity), and were free of musculoskeletal pain or injury during the study.

### Design of the Study

This study comprised three sessions separated by 24 to 48 h of rest. The first session familiarized participants with performing ballistic lower-limb extensions on the ergometer at high force-low velocity settings and vice-versa. The final two sessions were dedicated to assessing individual *F–v* and *P–v* relationships and each involved performing ballistic lower-limb extensions in 6 resistive conditions. Two sessions were planned to ensure that participants could maximize force production at very high velocities [e.g., 35].

### Ergometer

**Fig. 1:**
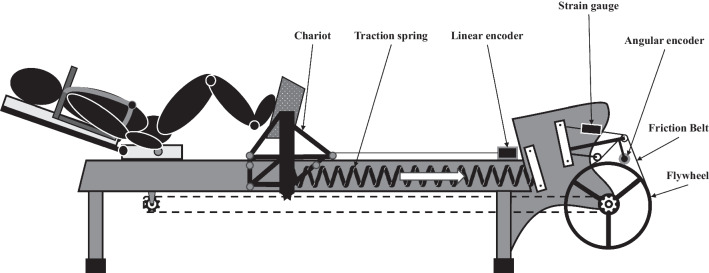
Schematic illustration of the instrumented horizontal leg-press ergometer

A shared limitation of previous works characterizing lower-limb acyclic force production is an inability to access extremely high velocities due mainly to the mechanical constraints imposed by the body weight and inertia. We addressed this issue by building an innovative instrumented leg-press ergometer (vide infra). The ergometer was a custom-build horizontal leg-press equipped with a flywheel surrounded by a friction belt (Fig. 1). It comprised of a metal frame supporting a fixed seat to which each participant was harnessed, with adjustable pads above their shoulders. Participants were positioned with their lower limbs flexed, and feet placed upon a chariot, in a position that approximated the bottom of a squat jump. The chariot was set on low-friction rails along which it was free to slide. In this manner, the ergometer allowed assessment of extensions without moving the entire body mass, where the user instead drove the chariot with the lower limbs. A friction belt and lateral traction springs provided control over resistance and assistance applied to the chariot motion, respectively, and enabled access to a broad range of mechanical conditions (notably, very high movement velocities). For each trial, the chariot was held in its starting position via electromagnets that were released by the participant via a hand-held button, allowing the chariot to move under ballistic intent (i.e., feet losing contact with the chariot at the end of the extension). For each lower-limb extension, participants were asked to apply force onto the chariot, which resulted in its acceleration and the concomitant acceleration of the flywheel linked by a chain. Instantaneous linear and angular displacements of the chariot and the flywheel were measured with linear (Kübler Group, Villingen-Schwenningen, Allemagne, 250 Hz) and angular (Baumer, Fillinges, France, 250 Hz) encoders, respectively. The friction forces applied by the belt on the flywheel ($F_{fb}$) was measured with a strain gauge (Futek, Irvine, USA, 250 Hz).

### Protocol

Each session began with a warm-up consisting of ~ 15 min of dynamic movements including sub-maximal and maximal unloaded squats, squat jumps and lower-limb ballistic extensions on the ergometer at high force-low velocity and vice-versa.

The first session focused on familiarizing athletes with the testing protocol. This included placing the participant in a position and adjusting the ergometer until they felt able to express maximum force. This placement was recorded for latter sessions and all subsequent trials. Participants then performed twenty to thirty ballistic lower-limb extensions on the ergometer in six different resistive and inertial conditions, interspersed with a minimum of 10 s passive rest periods, to habituate the participants with maximal effort (i.e., maximal neuromuscular activation) in each extension condition performed on the ergometer. To conclude, participants performed two maximum ~ 3-s maximum isometric contractions separated by 5 min of rest. Athletes were instructed to “push as hard and as fast as possible” for each trial, and verbally encouraged during the trial. The ergometer chariot was set in the previous self-selected preferred starting position (with knee and hip angle ranging from 72 to 114° and from 98 to 125°, respectively) with friction force set at maximum to prevent the chariot from any displacements on the rail.

The second and third sessions largely mirrored the first, except the resulting data were recorded to determine individual *F–v* and *P–v* relationships over the largest range of resistive conditions possible (in order of decreasing resistance): (1) resistive friction eliciting a movement velocity of ~ 0.3 m s^−1^, as the typical extension velocity observed during a one repetition maximum squat (C_1RM_; determined at the end of the familiarization session), (2) resistive friction corresponding to ~ 50% of maximal isometric force (C_50%Fmax_), only accelerating the chariot and the flywheel, (3) the friction belt being removed, without and (4) with two springs assisting the motion (C_ØFric-0S_ and C_ØFric-2S,_ respectively) and (5) only accelerating the chariot, the chain between chariot and flywheel being removed without and (6) with two springs in assistance (C_Char-0S_ and C_Char-2S_, respectively; Table 1). Two to three trials were performed for each resistive condition. For each trial, participants were asked to trigger the electromagnets and to hold lateral handles for upper-body stabilization, while producing as much force as possible and extending their lower limbs as fast as possible, aiming to push the chariot ballistically.

**Table 1:** Descriptive summary of the mechanical constraint and the force variables included in the computation, which was used to estimate the force developed over the lower limb push-off (Eq. 1) in the six resistive conditions

Condition	Description	Components included in force computation					
		$F_{{{\text{flywheel}}}}$	$F_{{{\text{chariot}}}}$	$F_{{{\text{friction}}}}$	$F_{{{\text{limbs}}}}$	$F_{{{\text{roll}}}}$	$F_{{{\text{spring}}}}$
C_1RM_	Acceleration of the chariot, the flywheel with friction, and the lower limbs	✓	✓	✓	✓	✓	×
C_50%Fmax_	Acceleration of the chariot, the flywheel with friction, and the lower limbs	✓	✓	✓	✓	✓	×
C_ØFric-0S_	Acceleration of the chariot, the flywheel (without friction), and the lower limbs	✓	✓	×	✓	✓	×
C_ØFric-2S_	Spring assisted acceleration of the chariot, the flywheel (without friction), and the lower limbs	✓	✓	×	✓	✓	✓
C_Char-0S_	Acceleration of the chariot and the lower limbs	×	✓	×	✓	×	×
C_Char-2S_	Spring assisted acceleration of the chariot and the lower limbs	×	✓	×	✓	×	✓

The signs « ✓» and « ×» correspond to the inclusion or the exclusion of the force component into the computation of the force developed by the lower limbs over the entire push-off, respectively. C_1RM_, Force developed at an extension velocity of ~ 0.3 m s^−1^; C_50%Fmax_, Force corresponding to 50% of isometric maximum; C_ØFric-0S_, Force produced while accelerating the chariot, the flywheel (without friction), and the lower limbs; C_ØFric-2S_, Force produced during spring-assisted acceleration of the chariot, the flywheel (without friction), and the lower limbs; C_Char-0S_, Acceleration of the chariot without flywheel and the lower limbs; C_Char-2S_, Spring assisted acceleration of the chariot (without flywheel) and the lower limbs; $F_{{{\text{flywheel}}}}$, force to accelerate the flywheel; $F_{{{\text{chariot}}}}$, force to accelerate the chariot;$F_{{{\text{friction}}}}$, the force to overcome the frictional forces applied by the belt on the flywheel;$F_{{{\text{limbs}}}}$, the force to accelerate the center of mass of the lower limbs; $F_{{{\text{roll}}}}$, the internal resistive force of the flywheel; *F*_spring_, the force of the tension springs

### Data Analysis

During isometric tests, force output was measured with the strain gauge on the friction belt and the maximal isometric force was calculated as the maximum averaged force over one second. During lower-limb extensions, as hip was fixed and feet were constantly in contact with the chariot, the instantaneous extension velocity (m s^−1^) and acceleration ($a_{{{\text{chariot}}}}$, in m s^−2^) of the lower limbs were determined as first- and second-order derivative of the low-pass filtered (20 Hz, Butterworth, 4th order) position signal obtained via the linear encoder. During each trial of all conditions, instantaneous force (in Newtons) was computed using Eq. 1 (detailed computations for each of the six resistive conditions in Table 1).

1 $$F = F_{{{\text{flywheel}}}} + F_{{{\text{chariot}}}} + F_{{{\text{friction}}}} + F_{{{\text{limbs}}}} + F_{{{\text{roll}}}} - F_{{{\text{spring}}}}$$

where *F*_flywheel_ is the force to accelerate the flywheel (Eq. 2), *F*_chariot_ to accelerate the chariot (Eq. 3), *F*_friction_ the force to overcome the frictional forces applied by the belt on the flywheel (Eq. 4), *F*_limbs_ the force to accelerate the center of mass of the lower limbs (Eq. 5), which was estimated from 2-D biomechanical model (detailed in the next paragraph), *F*_roll_ the internal resistive force of the flywheel (6.06 N) and *F*_spring_ the force of the tension springs (Eq. 6).

2 $$F_{{{\text{flywheel}}}} = \frac{I . \propto }{{d_{p} }}$$

3 $$F_{{{\text{chariot}}}} = m_{{{\text{chariot}}}} \cdot a_{{{\text{chariot}}}}$$

4 $$F_{{{\text{friction}}}} = F_{fb} \cdot \frac{{ d_{{{\text{flywheel}}}} }}{{d_{p} }}$$

5 $$F_{{{\text{limbs}}}} = m_{{{\text{limbs}}}} \cdot a_{{{\text{limbs}}}}$$

6 $$F_{{{\text{spring}}}} = n \cdot (k \cdot x + b)$$

where *I* is the moment of inertia of the flywheel (0.131 kg m^2^), ∝ (rad s^−2^) the instantaneous angular acceleration of the flywheel determined as the second-order derivative of the low-pass filtered (20 Hz, Butterworth, 4th order) position signal obtained from the angular encoder, *d*_*p*_ (m) the cog radius (0.032 m), *m*_chariot_ the mass of the chariot (15.15 kg) including the mass of the chain (1.05 kg), *d*_flywheel_ the radius of the flywheel (0.24 m) *m*_limbs_ the mass of the lower limbs, *α*_limbs_ (m s^−2^) the instantaneous acceleration of the lower limb’s center of mass estimated from a 2-D biomechanical model (see below), *n* the number of springs in assistance during the lower-limb extension, *k* the spring’s stiffness (320 N m^−1^), *b* the initial spring tension at free length (40 N) and *x* the instantaneous length of the spring determined from the instantaneous position of the chariot. Note that the rolling friction of the chariot on the rail was counterbalanced by the very low linear encoder traction force, and thus negligible (~ 0.01 N).

As proposed by Rahmani et al. [37] for the bench press exercise, the use of a simplified 2-D model with three segments is accurate enough to estimate center of mass displacement of the upper limbs. Thus, the 2-D model of the lower limbs used in the present study comprised three segments (thighs, shins and feet), with the length and mass of each estimated as a fraction of body height and mass, respectively [38]. The model allows for the determination of the center of mass instantaneous horizontal position of the three body parts during lower-limb extensions, as the barycenter of the thighs, shins and feet center of mass. Then, the center of mass instantaneous horizontal position of the lower limbs was estimated.

Force, velocity, and power were averaged over lower-limb extensions, which started when *a*_chariot_ became positive and ended when:

$$a_{{{\text{chariot}}}} < - \frac{{F_{{{\text{friction}}}} }}{{(m_{{{\text{chariot}}}} + m_{{{\text{limbs}}}} + m_{{{\text{flywheel}}}} )}}\quad {\text{or}}\quad a_{{{\text{chariot}}}} < 0$$

for conditions with the frictional forces on the flywheel, or for other conditions, respectively. Here, *m*_flywheel_ (126 kg) being the linear equivalent mass of the flywheel’s moment of inertia, which was computed as:

7 $$m_{{{\text{flywheel}}}} = \frac{I}{{d_{p}^{2} }}$$

For each participant, *F–v* and *P–v* relationships were determined from mean force, velocity and power values obtained from the best trial (i.e., highest mean power output) of the six different resistive conditions across all trials performed in the second and third sessions. These values were fitted with the basic first-order polynomial function to model a linear shaped individual *F–v* relationships (for the linear model) and with *Poly*_2_, *F&M’s*_Eq_ and *Hill’s*_Eq_ to model a curvilinear shaped individual *F–v* relationships (for the curvilinear model). As described by Hill in 1938, *Hill’s*_Eq_ corresponded to [22]:

8 $$F = \frac{{\left( {F_{0} + a} \right) \cdot b}}{v + b} - a$$

where *F* and *v* correspond to mean force and velocity over lower limb push-off, and *a* and *b* are constants. Accordingly to the Fenn & Marsh’s work published in 1935, *F&M’s*_Eq_ corresponded to [20]:

9 $$F = F_{0} e^{ - Av} - Bv$$

where *A* and *B* are constants.

The optimization procedure to fit the function of each model on the experimental force and velocity data consisted of applying of least squares method with polynomial regression for the basic first-order polynomial function and *Poly*_2_, or the Levenberg–Marquardt algorithm for *F&M’s*_Eq_ and *Hill’s*_Eq_. Optimizations were aiming to minimize the sum of squared errors, with the Levenberg–Marquardt algorithm set to 1.10^7^ model evaluations (i.e., number of evaluations of the loss function) and 1.10^6^ iterations (i.e., number of increments of the function’s variables). As the Levenberg–Marquardt algorithm finds only a local minimum of the loss function, which is highly dependent on the function’s starting parameters, the procedure of optimization was repeated one thousand times, considering at each repetition, a random starting value (within the range [0; + ∞] and [− 100; + ∞] for *Hill’s*_Eq_ and *F&M’s*_Eq_, respectively).

Individual *F*_0_ and *v*_0_ values were computed as the force- and velocity-axis intercept for each model. Individual *P–v* relationships were determined by integrating over velocity the *F–v* relationship. Then, *P*_max_ and *v*_opt_ were determined as the apex of the *P–v* relationship and the velocity condition at which *P*_max_ occurred, respectively. *F*_0_ and *P*_max_ were additionally expressed relative to body mass for (_Rel_*F*_0_ and _Rel_*P*_max_, respectively).

### Statistical Analysis

All data are presented as mean ± standard deviation (SD). To locate the mean force, velocity and power values obtained in the six resistive conditions, these outputs were expressed relative to *F*_0_, *v*_0_ and *P*_max_ obtained with the linear model (*F*_0-L,_ *v*_0-L_, and *P*_max-L_, respectively). When *Hill’s*_Eq_ was used in curvilinear models, the magnitude of the curvature of the *F–v* relationship was quantified by computing the ratio a/*F*_0_ [22].

The effect of fit function (the basic first-order polynomial function*,* *Poly*_2_, *F&M’s*_Eq_ and *Hill’s*_Eq_) on *F*_0_, _Rel_*F*_0_, *v*_0_, *P*_max_, _Rel_*P*_max_ and *v*_opt_ was tested with ANOVAs. These ANOVAs were performed after checking for normal distribution and sphericity with Shapiro–Wilk’s and Mauchly’s tests, respectively. If not met, a sphericity correction was applied. If the effect of the main factor was significant, Holm’s post hoc test was used to highlight significant differences. The magnitude of effect for each factor within the model was quantified via η^2^ and ω^2^, which were both interpreted as trivial, small, moderate and large when matching value of < 0.01, 0.01–0.06, 0.06–0.14 and > 0.14, respectively [39]. The magnitude of the difference (i.e., effect size; d) between outputs of the four functions (post-hoc tests) was reported via standardization to the between-subject standard deviation, as well as their associated confidence intervals. Effect sizes, d, were interpreted using qualitative thresholds, with < 0.2, 0.2 to < 0.6, 0.6 to < 1.2, 1.2 to < 2.0 and > 2.0 representing trivial, small, moderate, large, and very large effect, respectively [40]. For all statistical analyses, an alpha level of 0.05 was set.

To describe the GoF of the four functions, adjusted *r*^2^, SEE and distribution of residuals in force across velocity condition were computed. The magnitude of the differences between adjusted *r*^2^ and SEE from the four functions was assessed using specific scales as proposed by Rudsits et al. [41]. A clear improvement in adjusted *r*^2^ was identified when its value increased from one magnitude threshold to the next on the scale: 0.99, 0.92, 0.74, 0.50, and 0.20. This scale was also used to describe the magnitude of adjusted *r*^2^ corresponding to extremely high, very high, high, moderate, and low values, respectively. SEE values were compared using the qualitative thresholds above, but magnitude thresholds for assessing the standardized effect were halved [41].

Since SEE and residuals in force do not represent criterions for model selection with parsimony and statistical inferences, and interpreting change in adjusted *r*^2^ could be limited, models were compared using and Akaike Information Criterion (AIC) analysis (for details, please see [42]). Briefly, this method was used to detect whether *Poly*_2_, *F&M’s*_Eq_ and *Hill’s*_Eq_ lead to a great enough improvement of the GoF to justify their higher complexity (i.e., increased number degrees of freedom), in comparison to the first-order polynomial function (i.e., linear modelling). This analysis was conducted on each individual force and velocity data set. To perform this test, i) the sum of standard error of each model (SSE), ii) the corrected AIC index (AICc; used due to the ratio sample size/degrees of freedom being inferior to 40; Burnham and Anderson 2004), iii) the difference in AICc between the model with the smallest AICc and other models (ΔAICc), iv) the relative likelihood of each model, v) the AICc weight (AICc_w_) for each model and vi) the relative and vii) absolute AICc evidence ratio (AICc_w-ER_) were computed (for detailed definitions of these parameters, please see [43]).

## Results

Mean force, velocity and power developed over the push-off in the six resistive conditions are presented in Table 2, in raw values and expressed relative to *F*_0-L_, *v*_0-L_ and *P*_max-L_, respectively.

**Table 2:** Mean ± SD of mean absolute and relative force, velocity and power developed over lower-limb extension in the six resistive conditions, as well as the range of individual values in square brackets

	Force		Velocity		Power	
	N	%*F*_0-L_	m s^−1^	%*v*_0-L_	W	%*P*_max-L_
C_1RM_	1703 ± 325 [1373; 2337]	96.0 ± 3.6 [94; 99]	0.29 ± 0.08 [0.19; 0.46]	8.3 ± 1.9 [5;11]	492 ± 130 [274; 662]	31.4 ± 6.3 [19; 42]
C_50%Fmax_	1 490 ± 325 [1149; 2138]	82.0 ± 3.7 [76; 88]	0.67 ± 0.12 [0.50; 0.84]	19.0 ± 2.2 [16; 23]	971 ± 131 [800; 1173]	61.9 ± 5.7 [55; 70]
C_ØFric-0S_	1147 ± 155 [916; 1 410]	64.1 ± 6.7 [55; 75]	1.18 ± 0.10 [1.03; 1.30]	34.3 ± 5.6 [26; 41]	1366 ± 261 [998; 1816]	87.0 ± 8.8 [71; 94]
C_ØFric-2S_	1001 ± 134 [819; 1 161]	56.2 ± 7.7 [45; 66]	1.30 ± 0.07 [1.13; 1.39]	37.5 ± 4.7 [30; 45]	1299 ± 174 [1095; 1 547]	82.7 ± 4.6 [78; 90]
C_Char-0S_	628 ± 53 [536; 711]	35.0 ± 6.7 [24; 42]	2.27 ± 0.21 [1.95; 2.63]	65.0 ± 7.5 [56; 76]	1425 ± 188 [1214; 1732]	89.0 ± 9.9 [70; 100]
C_Char-2S_	465 ± 88 [369; 612]	26.6 ± 6.6 [16; 40]	2.67 ± 0.25 [2.38; 3.08]	76.6 ± 7.0 [64; 86]	1240 ± 262 [1055; 1842]	79.5 ± 13.4 [57; 100]

C_1RM_, Force developed at an extension velocity of ~ 0.3 m s^−1^; C_50%Fmax_, Force corresponding to 50% of isometric maximum; C_ØFric-0S_, Force produced while accelerating the chariot, the flywheel (without friction), and the lower limbs_;_ C_ØFric-2S_, Force produced during spring-assisted acceleration of the chariot, the flywheel (without friction), and the lower limbs; C_Char-0S_, Acceleration of the chariot without flywheel and the lower limbs; C_Char-2S_, Spring assisted acceleration of the chariot (without flywheel) and the lower limbs; N, Newtons; %*F*_0-L_, percentage of the force-axis intercept of the linear *F–v* relationship; m s^−1^, meters per second; W, watts; %*P*_max-L_, percentage of the apex of the *P–v* relationship, derived from the linear *F–v* relationship.

**Fig. 2:**
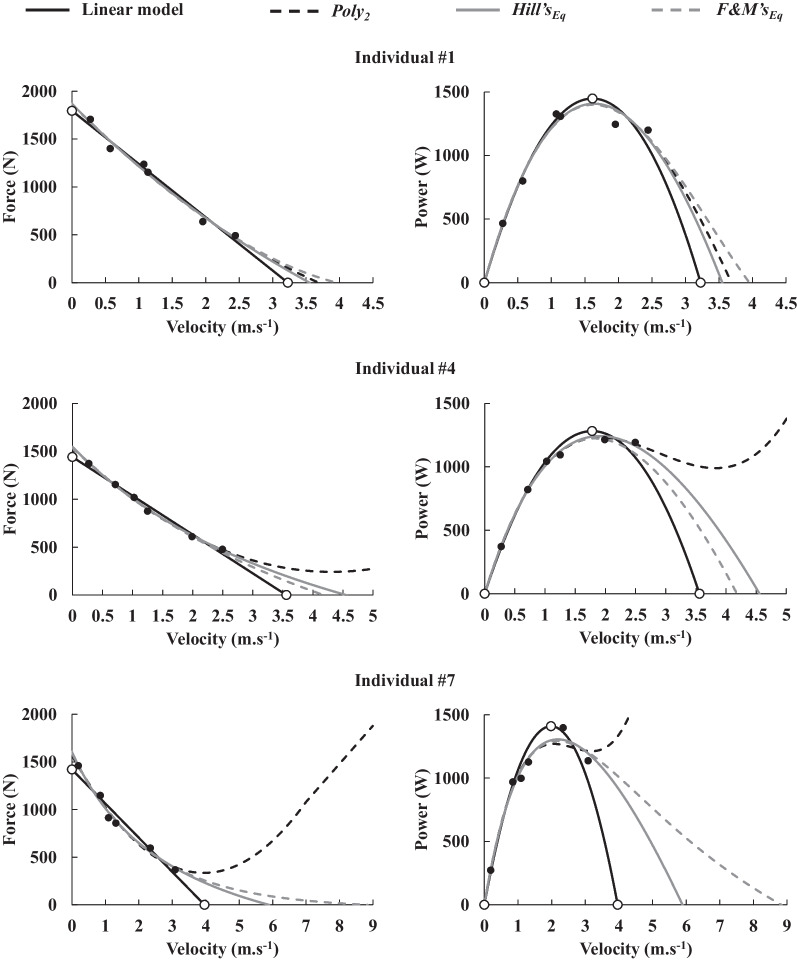
Three typical individual *F–v* relationships (left panels) drawn with the linear model (solid black lines), *Poly*_2_ (dashed black lines), *F&M’s*_Eq_ (dashed grey lines) and *Hill’s*_Eq_ (solid grey lines), and their resulting *P–v* relationship (right panels). Black points represent force, velocity and power data obtained from the six resistive conditions on the ergometer. The three individuals were chosen to represent typical examples of low (top panel; a/*F*_0_ = 2.70), moderate (middle panel; a/*F*_0_ = 1.57) and high (bottom panel; a/*F*_0_ = 0.53) curvature of the *F–v* relationship when using *Hill’s*_Eq_. *Poly*_2_, the second-order polynomial function; *Hill’s*_Eq_, Hill’s equation; *F&M’s*_Eq_, Fenn and Marsh’s equation

Typical examples of *F–v* relationships drawn using the linear and curvilinear models, associated with their resulting *P–v* relationship, are presented in Fig. 2. When using *Hill’s*_Eq_, a/*F*_0_ value was 1.06 ± 0.72 (no unit). *F*_0_, _Rel_*F*_0_, *v*_0_, *P*_max_, _Rel_*P*_max_ and *v*_opt_ values are presented in Table 3. When using *Poly*_2_, *v*_0_ could not be calculated for eight participants, due to the absence of an intercept with the velocity axis (i.e., the fit trended towards infinity; see examples of two individuals on Fig. 2, dashed black line on the left middle and bottom panels). There was a significant main effect of fit function on *F*_0_, _Rel_*F*_0_, *v*_0_, *P*_max_ and _Rel_*P*_max_ (all *p* < 0.05; η^2^ = 0.720, 0.786, 0.618, 0.785, 0.817, respectively, and ω^2^ = 0.047, 0.105, 0.271, 0.043 and 0.102, respectively), but not on *v*_opt_ (*p* = 0.380). Post-hoc analyses’ *p*-values and effect sizes are presented in the Table 3.

**Table 3:** Mean ± SD and the range of individual values of *F*_0_*,*_Rel_*F*_0_*,* *v*_0_*,* *P*_max_*,*_Rel_*P*_max_ and *v*_opt_ determined from the linear and curvilinear models, as well as effect sizes of the difference between models, associated to their interpretation and 95% confidence intervals in brackets

		*F*_0_	_Rel_*F*_0_	*v*_0_	*P*_max_	_Rel_*P*_max_	*v*_opt_
Linear model	Mean ± SD	1810 ± 339 N	25.6 ± 3.3 N kg^−1^	3.52 ± 0.54 m s^−1^	1570 ± 227 W	22.3 ± 1.7 W kg^−1^	1.76 ± 0.27 m s^−1^
	[range]	[1421–2438]	[21.0–31.3]	[2.84–4.70]	[1282–1923]	[20.1–24.7]	[1.42–2.35]
*Poly*_2_	Mean ± SD	1999 ± 418 N	28.2 ± 3.7 N kg^−1^	3.69 m s^−1^	1456 ± 212 W	20.7 ± 2.0 W kg^−1^	1.99 ± 0.82 m s^−1^
	[range]	[1539–2664]	[22.3–34.2]	x	[1231–1801]	[17.6–24.0]	[1.30–3.94]
	ANOVA	*p* < 0.001	*p* < 0.001	x	*p* < 0.001	*p* < 0.001	*p* = 0.817
	d	0.56	0.79	x	0.5	0.94	0.85
	(Interpretation)	(Small)	(Moderate)	x	(Small)	(Moderate)	(Moderate)
	[95%CI]	[0.77;0.35]	[0.55;1.03]	x	[0.32;0.68]	[0.64;1.25]	[0.76;2.46]
*Hill’s*_Eq_	Mean ± SD	2052 ± 461 N	28.9 ± 4.0 N kg^−1^	4.57 ± 0.84 m s^−1^	1471 ± 211 W	20.9 ± 1.9 W kg^−1^	1.84 ± 0.33 m s^−1^
	[range]	[1547–2716]	[22.3–34.8]	[3.55–5.91]	[1243–1812]	[18.4–24.2]	[1.52–2.53]
	ANOVA	*p* < 0.001	*p* < 0.001	*p* = 0.013	*p* < 0.001	*p* < 0.001	*p* > 0.999
	d	0.71	1	1.96	0.43	0.67	0.3
	(Interpretation)	(Moderate)	(Moderate)	(Large)	(Small)	(Moderate)	(Small)
	[95%CI]	[0.39;1.03]	[0.62;1.38]	[1.35;2.58]	[0.28;0.59]	[0.56;1.09]	[0.11;0.49]
*F&M’s*_Eq_	Mean ± SD	2029 ± 443 N	28.6 ± 3.9 N kg^−1^	5.22 ± 1.63 m s^−1^	1459 ± 216 W	20.7 ± 2.0 W kg^−1^	1.81 ± 0.35 m s^−1^
	[range]	[1535–2703]	[22.3–34.6]	[3.89–8.83]	[1224–1810]	[17.8–24.1]	[1.39–2.55]
	ANOVA	*p* < 0.001	*p* < 0.001	*p* < 0.001	*p* < 0.001	*p* < 0.001	*p* > 0.999
	d	0.65	0.91	3.17	0.49	0.82	0.18
	(Interpretation)	(Moderate)	(Moderate)	(Very large)	(Small)	(Moderate)	(Trivial)
	[95%CI]	[0.38;0.92]	[0.29;1.23]	[1.50;4.85]	[0.32;0.65]	[0.65;1.21]	[0.04;0.40]

*Poly*_2_, the second-order polynomial function; *Hill’s*_Eq_, Hill’s equation; *F&M’s*_Eq_, Fenn and Marsh’s equation; SD, standard deviation; d, Cohen’s effect size; 95%CI, 95% confidence intervals; N, Newtons; N kg^−1^, Newtons per kilogram; m s^−1^, meters per second; W, watts; W kg^−1^, watts per kilogram. Please note that only differences (i.e., *p*-values and d) between the linear and three other models are presented here

**Fig. 3:**
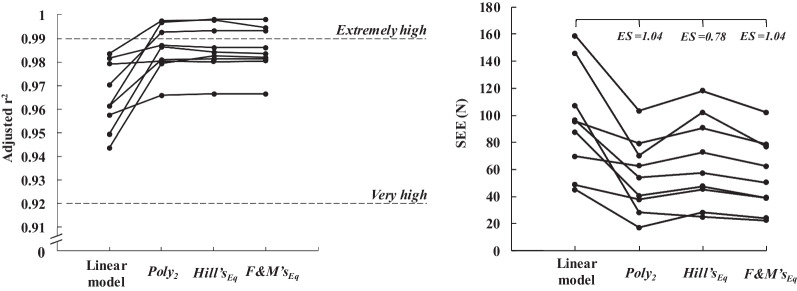
Differences in individual adjusted r^2^ and SEE for the linear and curvilinear models. Effect size (ES) of these differences are represented as threshold with their interpretation on the left panel (black dashed line and text) and as raw values on the right panel (black horizontal brackets and text). *Poly*_2_, the second-order polynomial function; *Hill’s*_Eq_, Hill’s equation; *F&M’s*_Eq_, Fenn and Marsh’s equation

**Fig. 4:**
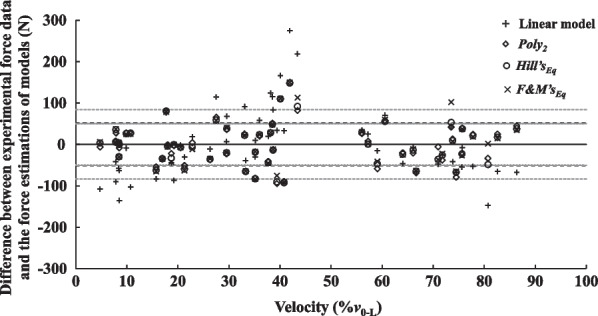
Distribution of residuals across participants presented according to the velocity condition. Residuals were computed as the difference between the force output during lower limb push-off in the 6 resistive conditions and the modeled force at the same velocity using the linear model (black plus signs), *Poly*_2_ (Black diamonds), *F&M’s*_Eq_ (black circles) and *Hill’s*_Eq_ (black crosses). SD of residuals for the linear model, *Poly*_2_, *Hill’s*_Eq_ and *F&M’s*_Eq_ are represented as dashed, dotted and full grey lines, and dashed black lines, respectively. *Poly*_2_, the second-order polynomial function; *Hill’s*_Eq_, Hill’s equation; *F&M’s*_Eq_, Fenn and Marsh’s equation

GoF of the linear and curvilinear models, assessed with adjusted r^2^, SEE and the distribution of force residuals on the velocity conditions spectrum, are presented as individual values on the panels of Figs. 3 and 4. Effect size of change in SEE were large, when comparing the linear model to the curvilinear models, but a clear improvement of adjusted r^2^ was observed for only three participants (Fig. 3, left panel). Comparisons of the linear and curvilinear models using AICc analysis are presented in Table 4.

**Table 4:** Mean ± SD and individual values in brackets of SSE, AICc, ΔAICc, AICc_w_, as well as absolute and relative AICc_w-ER_, associated to their respective interpretations

	Interpretation	Linear model	*Poly*_2_	*Hill’s*_Eq_	*F&M’s*_Eq_
SSE (N^2^)	Sum of squared errors	40,581 ± 32 443 [8111–100 708]	14,395 ± 13,278 [1150–42547]	14 507 ± 13,311 [1980–41599]	15,380 ± 14,256 [1846–41855]
AICc	Index of information lost by approximating the observed data (Kullback–Leibler estimate). Lower values represent less lost information and proximity to “reality”	69.054 ± 5.244 [61.255–76.369]	91.884 ± 6.825 [79.535–101.200]	92.031 ± 6.487 [82.793–101.064]	92.188 ± 6.754 [82.373–101.101]
ΔAICc	Difference to the best model AICc. Allows for model ranking and assessing relative performance	Ø	22.830 ± 4.822 [13.953–28.738]	22.977 ± 5.302 [11.124–28.682]	23.134 ± 5.457 [10.703–28.794]
AICc_w_	Relative weight of evidence for each model, as the probability for being the best model for the observed data, given the candidate set of models	0.999 ± 0.003 [0.991–1.000]	0.000 ± 0.000 [0.000–0.001]	0.000 ± 0.001 [0.000–0.004]	0.001 ± 0.002 [0.000–0.005]
AICc_w-ER_	Quantification of the strength of evidence in favor of best model. Practically, *“how* *much* *less* *likely* *the* *model* *is* *than* *the* *best model?”*	Ø	435,608 ± 611,109 [1071–1739075]	446,844 ± 599,662 [260–1690866]	472,653 ± 621,639 [211–1788623]
AICc_w-ER_ (%)	Ratio of AICc_w_ of the compared model to the AICc_w_ of the best model, corresponding to a normalized probability that the best model is to be preferred	Ø	99.988 ± 0.031 [99.907–100.000]	99.956 ± 0.127 [99.617–100.000]	99.947 ± 0.157 [99.528–100.000]

*Poly*_2_, the second-order polynomial function; *Hill’s*_Eq_, Hill’s equation; *F&M’s*_Eq_, Fenn and Marsh’s equation.

## Discussion

An innovative leg-press ergometer allowed lower-limb external force production measurements over very broad mean extension velocity range (individual values ranged from ~ 0.2 to ~ 3.1 m s^−1^). Expressed relative to individual force–velocity relationships, the range corresponded to ~ 8 and ~ 77%*v*_0-L_ (individual values ranged from ~ 5 to ~ 86%*v*_0-L_). Over the extended range of experimental data, following the principle of parsimony, the linear model was very likely the best model to describe the force–velocity relationship, compared with curvilinear alternatives.

The novel ergometer presented here allows ballistic and horizontal (i.e., without the resistance of the body weight) lower-limb extensions, with assistance to the motion, without upper-body movement and with low external masses (i.e., only lower limb and chariot masses). Where the methodological settings (e.g., technology) of previous studies allowed ‘high’ velocity conditions of only ~ 1.7–2 m s^−1^ [13, 17, 29, 44], the ergometer made it possible to reach mean extension velocities of up to 2.7–3 m s^−1^ (Table 2). Such high velocity conditions were only accessible due to a combination of both very low resistive and inertial conditions and technical assistance, since the best individual in C_Char-0S_ showed similar value as the mean of individual in C_Char-2S_. In acyclic lower-limb extensions, reaching very high movement velocity is challenging since each effort starts at zero velocity and requires the inertia of the moving masses to be overcome in each resistive/loading condition. In contrast, up to 90%*v*_0-L_ is attainable without cumbersome methods and equipment in cyclic movements (e.g., running and cycling), because high-velocity lower-limb extensions occur when the moving masses have been already accelerated [11, 33].

In the present study, linear and curvilinear models showed equally distributed residuals across velocity conditions (i.e., 5–86%*v*_0-L_ range) and small SDs within 100 N (Fig. 4). These results highlight that all models describe the force and velocity data over the tested experimental range with similar precision. Further, each model provided a very to extremely high-quality fit, with low SEE (Fig. 3). Change in SEE from the linear model to curvilinear models were large with a clear improvement of adjusted r^2^ observed for only three participants. This was mainly caused by isolated cases of high errors for the linear model (Fig. 4). Overall, even if curvilinear models fit the data minorly better compared to the linear model [e.g., 17, 25, 27], the GoF of all models was in the high to very high quality range. Nevertheless, when examining the prediction error and relative quality of the different models (i.e., AIC, see Table 4) the linear model had a ~ 99% chance to be the best model and displayed extremely strong evidence in its favor. Consequently, despite higher GoF of curvilinear models, their higher degrees of freedom did not improve accuracy of *F–v* relationship description to an extent warranting their utilization. This follows the principle of Occam’s razor, which states that among models with similar accuracy, the one with the fewest assumptions and parameters is preferable [34]. These results support using simpler linear model to describe the *F–v* relationship in acyclic lower-limb extensions over a broad range of resistive conditions.

The validity of a model describing the *F–v* relationship relies on the practical and physiological relevance of its output parameters – here corresponding to *F*_0_, *v*_0_, *P*_max_ and its slope. In the present study, *P*_max_ values estimated from the linear model were very close to the experimental power output measured around *v*_opt_ (e.g., right bottom panel, Fig. 2). These findings are in line with previous studies, which reported similar results on leg-press and horizontal and vertical squat jumps [14, 25, 29]. Consequently, *P*_max_ values estimated from the linear model are likely accurate estimates of true values and are physiologically relevant. Comparatively, curvilinear models exhibited lower *P*_max_ values than the linear model by ~ 100–120 W (Table 3 and Fig. 2), and these values can be lower than experimental power output measured around *v*_opt_ (e.g., right bottom panel, Fig. 2). Thus, despite the values being rational, curvilinear models probably underestimate *P*_max_. These results contrast reports of higher *P*_max_ estimated by curvilinear models (using *Poly*_2_ and *Hill’s*_Eq_ [25, 45]), which might be explained by these studies lacking experimental data beyond 50%*v*_0_—leading to higher estimations of *v*_0_, and accordingly *P*_max_. Nevertheless, the linear and curvilinear models appear to provide comparable estimates of *P*_max_ when including additional resistive/loading conditions around *v*_opt_ [25]. In the present work, *F*_0_ estimated from the linear model was slightly to moderately lower compared to estimations of curvilinear models (Table 3 and Fig. 2), and both congruent with typical maximum strength values (e.g., ~ 1.5 to 1.8 times body mass for half-squat 1-RM). This is in line with a previous study where a curvilinear model (using *Hill’s*_Eq_) estimated higher values of *F*_0_ in comparison to the linear model [45]. Nevertheless, curvilinear models can provide quasi-similar values of *F*_0_ when including additional resistive/loading conditions in the high force-low velocity domain, notably close to *F*_0_ [25, 45]. Overall, linear and curvilinear models provide physiologically coherent *F*_0_ and *P*_max_ values, but the precision of the latter relies on sufficient velocity conditions (i.e., longer testing duration) to avoid over- or underestimation.

Where *P*_max_ and *F*_0_ values appear similar across models, *v*_0_ values extrapolated from linear and curvilinear models can diverge strikingly. In the same manner as with *F*_0_ and *P*_max_, one clear means of testing the physiological relevance of *v*_0_ is measuring lower-limb force production at extremely high velocities (i.e., close to the graphical intercept) and comparing the values. Nonetheless, despite the very high extension velocities attained in this study (Table 2), substantial differences in *v*_0_ persisted between the linear model and curvilinear models (from ~ 4 to ~ 50%, Table 3). In the present study, the linear model estimated *v*_0_ values of ~ 3.2 m s^−1^. These values are comparable to estimations of *v*_0_ with a linear model during a simulated leg-press task including force collection from ~ 5 to 90%*v*_0_ [32]. In addition, comparable values can be estimated from acyclic mono-articular knee extensions under very low resistive and inertial conditions [12, 21] by applying the 2-D model previously mentioned (~ 650 and 750°/s, corresponding to lower-limb extension linear velocities of ~ 2.5 and 3 m s^−1^, respectively). Finally, theoretical maximal pedaling cadences for active individuals ( ~ 230 rotations per minute; Dorel et al. 2010) and experimental maximal pedaling cadences of elite track cyclists (~ 270–300 rotations per minute) would correspond (considering a typical crank length of 0.175 m) to lower-limb extension velocities of ~ 2.7 m s^−1^ and ~ 3.2–3.5 m s^−1^, respectively. Overall, these values are in line with *v*_0_ values estimated here from the linear model (Table 3) and lower than those extrapolated here from curvilinear models. Thus, although the compared lower-limb extension linear velocities are representative of individuals with different anthropometrics and training histories, and were measured using different movements, their proximity to *v*_0_ values estimated from the linear model support the physiological relevance of the latter. Furthermore, they highlight the potential overestimation of *v*_0_ values estimated by curvilinear models in acyclic lower extensions; this overestimation is important to consider when evaluating the *F–v* relationship with a narrow range of velocity conditions (e.g., ~ 20–60%*v*_0_), the likes of which are common in field testing, since *v*_0_ values are more likely to be overestimated. In this context, the linear model should be preferred to avoid estimations of potential erroneous values. A critical limitation of curvilinear models is the potential for values that are not physiologically plausible [25]. For example, the curvilinear model including *Poly*_2_ exhibited the lowest SEE of all curvilinear models (Fig. 3, right panel), but did not define *v*_0_ for 8 out of 9 participants (e.g., left middle and bottom panels, Fig. 2). If taken at face value, the practical interpretation for these athletes is that their force production at very high velocities greatly exceed that at low velocities—trending toward infinite force capabilities. This interpretation is nonsensical, and supports the argument that higher GoF of a model does not systematically lead to more accurate and valid outcomes. Consequently, the most appropriate model should be selected per its ability to describe at best the properties of the system studied (e.g., the human biological features of external force production capabilities during a multi-joint movement), rather than solely according to the mathematical function which fits at best the experimental data.

It is important to note that, even if the true *F–v* relationship in acyclic lower-limb extensions were non-linear beyond ~ 86%*v*_0-L_, it would not challenge the application of the linear model within the range 0–86%*v*_0-L_. Indeed, this range represents most of the practical field situations, with the linear extrapolation of *F*_0_ and *v*_0_ representing the theoretical limits of the neuromuscular system. Thus, the use of the linear model within this ~ 86% range does not challenge scientific applications in performance, testing and training related to the linear shaped *F–v* relationship in multi-joint movements [e.g., 9, 46]. Consequently, practitioners and coaches should be confident in using field approaches, while acknowledging their accuracy is reliant on various methodological factors and rigorous measurements.

Beyond the GoF of a model and the physiological relevance of its output parameters, the reliability of the latter is also a key point to test the quality of a model. High reliability has been often reported for *F*_0_ and *P*_max_ and moderate to high reliability for *v*_0_, when estimated from the linear model [17, 47–49]. Only one study has compared the reliability of linear and curvilinear models (using the basic exponential function and *Poly*_2_), which showed similar (*F*_0_) and lower (*v*_0_ and *P*_max_) reliability for curvilinear models over an assessed range of ~ 10–50%*v*_0-L_ [25]. However, it is important to note that such restricted ranges of velocity conditions will very likely reduce the reliability of the estimated parameters, especially from complex models, since they are more likely to vary with measurement error. Consequently, even if the linear model seems to yield greater reliability, further studies using a wider range of experimental conditions are needed. While determination of models’ outputs reliability in this study wasn’t feasible, inter-trial reliability indicate coefficient of variation scores fell within acceptable ranges of 1.6 and 5.8% for mean force and velocity across inertial/resistive/assisted conditions, considering 4—8 participants.

Finally, differences in models used to describe the *F–v* relationship between acyclic lower-limb extensions and during single-joint or in vitro single-muscle contraction have been supported by the fact that the former refers to external rather than intrinsic muscle force production. Indeed, the former involves specific underlying mechanisms, including neural control of various muscle groups, activation and segmental dynamics, which are not all encompassed in the two latter conditions [1, 14, 32]. In this sense, Bobbert [32] reported a “quasi-linear” *F–v* relationship over a wide range of simulated velocity conditions (~ 5–90%*v*_0-L_) in acyclic lower-limb extensions, despite using Hill’s curvilinear equation to characterize intrinsic force production capabilities of individual muscles. Furthermore, the linearity of the *F–v* relationship in acyclic lower-limb extensions is in line with the linearity observed in other multi-joint movements, such as cycling and running, where lower limb force production was measured over a wide range of velocity conditions, notably on the velocity end (i.e., ~ 20–90%*v*_0-L_ [11, 33]). Consequently, biomechanical simulations and studies on other multi-joint movements tend to align with linear modelling on *F–v* relationship obtained in acyclic lower-limb extensions.

## Perspectives

The unique design of the ergometer used in this study allowed lower-limb force production measurements from very low (similar to one-repetition maximum) to very high (approaching estimates of physiological maximums) velocity conditions. Similar devices that can generate comparable conditions could provide a means of targeting the development of force produced at very high, and otherwise inaccessible velocities during training; this is particularly interesting for weak population to train their specific deficit in velocity capabilities [50]. This type of design allows the force–velocity relationship to be evaluated i) without carrying external loads, which may be safer notably for frail populations, and ii) on a wide range of velocity conditions, which could increase the accuracy and the reliability of *v*_0_ and *P*_max_ [51]. When examining a greatly expanded range of velocity conditions the linear model was the most appropriate to describe the force–velocity relationship in acyclic lower-limb extension. Since most field situations occur within the explored range, actual testing and training methods applying such a model to multi-joint movements are justifiable [e.g., 52, 53].

## Conclusion

Very high lower limb extension velocities can be reached using a specialized leg-press (assisted horizontal acyclic lower limb extensions without moving the rest of the body). The implementation of such an ergometer allowed a much larger portion of the force–velocity relationship to be examined than previously accessible. Over this wide range, the force–velocity relationship appeared well described by the linear model, since curvilinear models did not improve accuracy to a degree warranting their utilization. Moreover, where curvilinear models can produce irrational outputs (e.g., *v*_0_) under typical testing settings, the linear model has provided physiologically appropriate values. With this in mind, practitioners should feel confident in adopting linear modelling when assessing the force production capabilities of the lower limbs at different velocities during acyclic ballistic extensions. Technical and methodological improvements of the ergometer could potentially help further widen the range of accessible velocity conditions and test the linearity of the force–velocity relationship in velocity conditions close to the maximal extension velocity.

## Data Availability

The data set supporting the conclusions of this article will be made available by the authors on reasonable request.

## Abbreviations

95% CI: 95% Confidence intervals
%*v*_0_: Percentage of the velocity-axis intercept of the *F–v* relationship
%*F*_0_: Percentage of the force-axis intercept of the *F–v* relationship
%*v*_0-L_: Percentage of the velocity-axis intercept of the linear *F–v* relationship
%*F*_0-L_: Percentage of the force-axis intercept of the linear *F–v* relationship
%*P*_max-L_: Percentage of the apex of the *P**–**v* relationship, derived from the linear *F–v* relationship
$\propto$: The instantaneous angular acceleration of the flywheel
AIC: Akaike Information Criterion
$a_{{{\text{chariot}}}}$: The instantaneous acceleration of the feet support
$a_{{{\text{limbs}}}}$: The instantaneous acceleration of lower limb’ centre of mass
*C*_ØFric-2S_: Condition of lower limb extension, during which only the feet support and the flywheel of the ergometer is accelerated with the assistance of 2 springs
*C*_ØFric-0S_: Condition of lower limb extension, during which only the feet support and the flywheel of the ergometer is accelerated with no spring assistance
C1RM: Condition of lower limb extension performed against resistive frictional force close to the maximal isometric force, leading to extension velocity close a one-repetition maximum’s typical performed velocities
C_50%Fmax_: Condition of lower limb extension performed against resistive frictional force corresponding to ~ 50% of maximal isometric force
C_Char-2S_: Condition of lower limb extension during, which only the feet support of the ergometer is accelerated with the assistance of 2 springs
C_Char-0S_: Condition of lower limb extension during, which only the feet support of the ergometer is accelerated with no spring assistance
$d_{{{\text{flywheel}}}}$: The radius of the flywheel
$d_{p}$: The cog radius of the ergometer
d: Effect size
$F$: The instantaneous force developed by lower limbs during extension
*F*_0_: The theoretical maximal force that lower limbs could produce over one extension at zero velocity
*F&M*’s_Eq_: Fenn and Marsh’s equation
$F_{{{\text{chariot}}}}$: The force developed by lower limbs to accelerate the feet support of the ergometer
$F_{fb}$: The friction force applied by the belt on the flywheel of the ergometer
$F_{{{\text{limbs}}}}$: The force developed by lower limbs to accelerate its own mass
$F_{{{\text{flywheel}}}}$: The force developed by lower limbs to accelerate the flywheel
$F_{{{\text{friction}}}}$: The force developed by lower limbs to overcome the frictional force applied by the belt on the flywheel
$F_{{{\text{roll}}}}$: The internal resistive force of the flywheel
$F_{{{\text{spring}}}}$: The force produced by springs in assistance to the movement
*F*–*v*: Force-velocity
GoF: Goodness of fit
Hill’s_Eq_: Hill’s equation
$I$: The moment of inertia of the flywheel
m s−1: m per second
$m_{{{\text{chariot}}}}$: The mass of feet support
$m_{{{\text{limbs}}}}$: The mass of lower limbs
$m_{{{\text{flywheel}}}}$: The linear equivalent mass of the flywheel’s moment of inertia
*N*: Newtons
*P*_max_: The maximal power capacity at the optimal extension velocity
*Poly*2: the second-order polynomial function
*P*-*v*: Power-velocity
*r*^2^: coefficient of determination
SD: standard deviation
SEE: standard error of estimate
*v*_opt_: optimal velocity
*v*0: The theoretical maximal velocity until which lower limbs could produce force over one extension
*W*: Watts
